# A Cationic Smart Copolymer for DNA Binding

**DOI:** 10.3390/polym9110576

**Published:** 2017-11-04

**Authors:** Tânia Ribeiro, Ana Margarida Santiago, Jose Manuel Gaspar Martinho, Jose Paulo Farinha

**Affiliations:** CQFM—Centro de Química-Física Molecular and IN—Institute of Nanoscience and Nanotechnology, Instituto Superior Técnico, Universidade de Lisboa, 1049-001 Lisboa, Portugal; tania.ribeiro@tecnico.ulisboa.pt (T.R.); anasantiago.silva@gmail.com (A.M.S.); jgmartinho@tecnico.ulisboa.pt (J.M.G.M.)

**Keywords:** block copolymer, stimuli responsive polymer, DNA, fluorescence, FCS

## Abstract

A new block copolymer with a temperature-responsive block and a cationic block was prepared by reversible addition-fragmentation chain transfer (RAFT) polymerization, with good control of its size and composition. The first block is composed by di(ethylene glycol) methyl ether methacrylate (DEGMA) and oligo(ethylene glycol) methyl ether methacrylate (OEGMA), with the ratio DEGMA/OEGMA being used to choose the volume phase transition temperature of the polymer in water, tunable from ca. 25 to above 90 °C. The second block, of trimethyl-2-methacroyloxyethylammonium chloride (TMEC), is positively charged at physiological pH values and is used for DNA binding. The coacervate complexes between the block copolymer and a model single strand DNA are characterized by fluorescence correlation spectroscopy and fluorescence spectroscopy. The new materials offer good prospects for biomedical application, for example in controlled gene delivery.

## 1. Introduction

Stimuli-responsive polymers (SRP), also known as “smart” polymers, change their properties, such as chain conformation, interactions and aggregation state, in response to external stimuli (temperature, pH, pressure, ionic strength, light, etc.) [1]. The versatility in the preparation of SRPs with different comonomers, sizes, and architectures have originated a new class of materials, with promising applications in many areas, from sensors to environmental remediation, smart catalysts, nanoreactors, multiresponsive coatings, and in particular, in the biomedical field [2,3,4,5]. 

Polymers that are temperature-responsive in water, collapsing when the temperature increases, are probably the most studied SRPs. For these polymers, the balance between segment−segment and segment−solvent interactions can be shifted by changing the temperature, inducing a reversible volume phase transition (VPT) at a certain temperature. Below this volume phase transition temperature (*T*_VPT_) the chains are in a solvated coil conformation, while above the *T*_VPT_ they adopt a collapsed globule conformation that results from the balance between the hydrophobic interactions between polymer segments and the hydrogen bonding with water. Among temperature-responsive polymers, poly(*N*-isopropylacrylamide) (PNIPAM) is the most commonly used [6,7], probably because its *T*_VPT_ of 32 °C is close to physiological temperatures. More recently, polymers based on ethylene glycol methacrylate derivatives (PEG-methacrylates) have emerged as good candidates to substitute PNIPAM. These copolymers are biocompatible, have good water solubility below the *T*_VPT_, and feature a reversible VPT at a temperature that can be tuned by adjusting the molar ratio between monomers with different numbers of ethylene glycol units [8,9,10,11,12,13]. Due to their low toxicity, these copolymers have a great potential for use in biomedical applications [9].

Cationic polymers have been used as non-viral nanocarriers to deliver DNA to cells for therapeutic proposes [14,15,16]. In this application, it is important to control the binding of the DNA strands to the polymer, as well as their release [17,18]. The main advantages of these vectors are their safety, greater flexibility and more facile manufacturing when compared to the viral vectors. 

The design of non-viral nanocarriers with DNA transfer effectiveness comparable to viral vectors, is an important topic in the development of these polymers.

**Figure 1:**
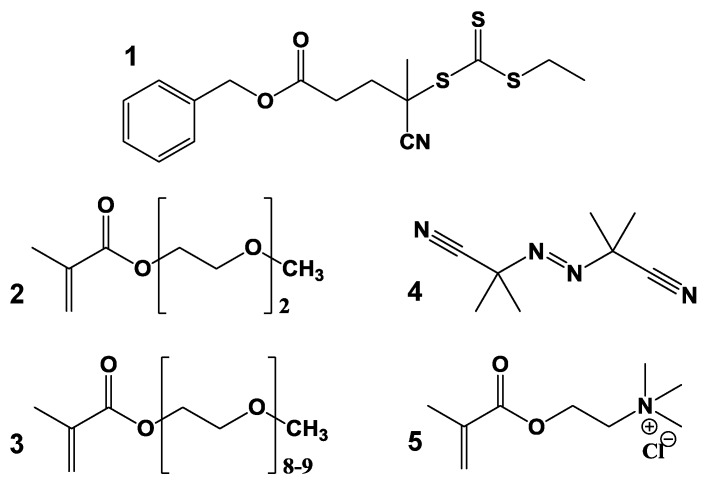
Structures of the reversible addition-fragmentation chain transfer (RAFT) chain transfer agent (CTA, **1**); the monomers di(ethylene glycol) methyl ether methacrylate (DEGMA, **2**), oligo(ethylene glycol) methyl ether methacrylate (OEGMA, **3**) and trimethyl-2-methacroyloxyethylammonium chloride (TMEC, **5**); and the initiator 2,2′ Azobis(isobutyronitrile) (AIBN, **4**).

In this work, we prepared a new SRP with a PEG–methacrylate thermoresponsive block and a cationic block. By incorporating the charged block, we increase the functionality of the smart material, for DNA binding, with possible application in gene delivery without significantly increasing their cytotoxicity [19,20,21,22]. The thermoresponsive block can be tuned to trigger the release of the DNA chain. The block copolymers were synthesized by reversible addition-fragmentation chain transfer (RAFT) polymerization, with a high degree of control over the size and composition of the copolymer. The first block of the copolymer is composed by di(ethylene glycol) methyl ether methacrylate (DEGMA, **2**, Figure 1) and oligo(ethylene glycol) methyl ether methacrylate (OEGMA, **3**, Figure 1). The ratio of DEGMA/OEGMA, as well as the degree of OEGMA polymerization, can be used to tune the *T*_VPT_ of this block from ca. 26 to above 92 °C [23,24]. The cationic block is composed by trimethyl-2-methacroyloxyethylammonium chloride (TMEC, **5**, Figure 1) units positively charged at physiological pH values, and so adequate for binding DNA strands by strong electrostatic interactions. These interactions lead to the formation of stable coacervate structures between a model DNA strand and the thermoresponsive cationic copolymer. 

To study the polymer-DNA coacervate structures we rely on fluorescent techniques, which offer very high sensitivity and selectivity, allowing not only the detection of fluorescent components in mixtures or assemblies, but also the study of the systems themselves by following the changes in emission properties of the fluorescence components [25]. In this work, fluorescently-labeled DNA single strands were used to determine the size of the polymer-DNA coacervated complexes by Fluorescence Correlation Spectroscopy (FCS), and access their stability and evolution by fluorescence spectroscopy. The temperature-responsive copolymer is shown to form stable complexes with single strand DNA oligomers, with promising biomedical applications.

## 2. Materials and Methods

### 2.1. Materials

Di(ethylene glycol) methyl ether methacrylate (**2**, DEGMA, 95%), oligo(ethylene glycol) methyl ether methacrylate (**3**, OEGMA) and trimethyl-2-methacroyloxyethylammonium chloride (**5**, TMEC, ca. 80% in water) were purchased from Sigma-Aldrich (St. Louis, MO, USA) and passed through a basic aluminum oxide column to remove inhibitors prior to use. 2,2′ Azobis(isobutyronitrile) (**4**, AIBN) was purchased from Wako Chemicals (Richmond, VA, USA) and crystallized twice from methanol prior to use. 1,4-Dioxane, *N*,*N*-dimethylformamide (DMF) and diethyl ether from Sigma-Aldrich were used without further purification. The synthesis of benzyl 4-cyano-4-(ethylthiocarbonothioylthio)-pentanoate (**1**, CTA) was previous reported [23]. Poly-thymine oligonucleotides (dT_25_) and fluorescent poly-thymine oligonucleotides labelled with rhodamine X (dT_25_-ROX), both with 25 thymine units, were purchased from Thermo (Darmstadt, Germany) in the lyophilized form (HPLC grade). Milli-Q water, from a Millipore system, with a resistivity of 18.2 MΩ·cm was used for samples preparation.

### 2.2. Synthesis of the Thermoresponsive Block of the Copolymer by RAFT Polymerization

A mixture of di(ethylene glycol) methyl ether methacrylate (**2**, 1.68 g, 8.93 mmol), oligo(ethylene glycol) methyl ether methacrylate (**3**, 475.56 mg, 1 mmol), benzyl 4-cyano-4-(ethylthiocarbonothioylthio)-pentanoate (**1**, 26.49 mg, 0.075 mmol), and 2,2’ Azobis(isobutyronitrile) (**4**, 2.50 mg, 0.015 mmol) in 7.5 mL of 1,4-dioxane was purged with nitrogen for 30 min. The solution was stirred at 70 °C overnight. The polymer was then isolated and purified by repeated precipitation in cold diethyl ether and dried under vacuum for 24 h. The molecular weight and polydispersity index were obtained by size exclusion chromatography (SEC) and characterized by ^1^H-NMR (CDCl_3_): δ (ppm) 0.88, 1.04 (m, 186H CH_3_–C_4°_); 1.53–2.11 (m, 120H C_4°_–CH_2_–C_4°_); 3.39 (s, 186H–O–CH_3_); 3.48–3.81 (m, 354H–O–(CH_2_–CH_2_–O)_n_–); 4.10 (m, 120H–CO–O–CH_2_–); 5.12 (s, 2H–CH_2_–Ar); 7.35 (m, 5H–Ar).

### 2.3. Synthesis of the Charged Block of the Copolymer by RAFT Polymerization

The second block was synthesized using the ionic monomer trimethyl 2-methacroyloxyethylammonium chloride (**5**). A mixture of the first block polymer chains (300.68 mg), AIBN (0.257 mg, 0.002 mmol), and TMEC (**5**, 122.20 mg, 0.588 mmol) in 1.4 mL of DMF was purged with nitrogen for 30 min. The solution was stirred at 70 °C overnight. The block copolymer chains were isolated and purified by repeated precipitation in cold diethyl ether and then dried under vacuum for 24 h. Elemental analysis was used to determine the molecular weight of the block copolymer.

### 2.4. Preparation of Block Copolymer-DNA Coacervates

Solutions of the stimuli-responsive block copolymer (SRP) and fluorescent oligonucleotides were prepared in water. SRP:DNA and SRP:DNA-ROX samples correspond to the mixtures of the block copolymer with the dT_25_ oligonucleotide and the Rhodamine-labeled oligonucleotide (dT_25_-ROX), respectively. These mixtures were prepared with 1:1, 2:1, and 4:1 molar ratios of SRP:DNA or SRP:DNA-ROX. All mixtures were stirred for 30 min at 25 °C before measurements.

### 2.5. Size Exclusion Chromatography (SEC)

Aqueous size exclusion chromatography (SEC) was performed in a Shimadzu system (Kyoto, Japan) comprising a DGU-12A solvent degasser, a LC-10AT pump, a CTO 10A column oven, a RID-10A refractive index detector and a SPD-10A Shimadzu UV–Vis detector, using a flow rate of 1 mL/min. A PL 5.0 mm bead-size guard column (50 × 7.8 mm^2^) was used before three PL aquagel–OH columns (50, 40, 30; 8 μm). Calibration was performed with PEO standards from 500 to 500,000 g/mol. SEC analysis of the polymers were also performed in *N*,*N*-dimethylacetamide (DMAc; 0.03% *w*/*v* LiBr; 0.05% 2,6-di-butyl-4-methylphenol (BHT)) at 50 °C and a flow rate of 1 mL/min, using a Shimadzu system (Kyoto, Japan) comprising a SIL-10AD auto-injector, a PL 5.0 mm bead-size guard column (50 × 7.8 mm^2^) and four linear PL (Styragel) columns (105, 104, 103, and 500 Å), with a RID 10A differential refractive-index detector.

### 2.6. Nuclear Magnetic Resonance (NMR)

^1^H-NMR spectra were recorded in a Bruker ACF300 (300 MHz) spectrometer (Billerica, MA, USA) using CDCl_3_ as solvent. Ethylene glycol monomer conversion was determined by comparing the vinyl proton signal (~5.6–6.1, 2H/mol ethylene units in the copolymers) to the total methylene groups attached to the methacrylate signal (~4.2 2H/mol ethylene units in the copolymers).

### 2.7. Dynamic Light Scattering (DLS)

The hydrodynamic diameter of the block copolymer chains and SRP:DNA mixtures were obtained using a Brookhaven Instruments (Brookhaven, NY, USA) equipment with a He–Ne laser (35 mW, 632.8 nm, Spectra Physics, model 127), an avalanche photodiode detector, a BI-200SM goniometer and a BI-9000AT correlator. The autocorrelation functions were analysed by Laplace inversion (CONTIN: BI-ZP, software package, Brookhaven, NY, USA). The measurements were carried out on glass cylindrical cells to simplify the corrections needed for refractive index variations, using a circular vat cell containing decaline to minimize light refraction. The samples were prepared in water, stirred 30 min and filtered with 0.2 μm cellulose acetate filters prior the measurement. The samples equilibrated during 15 min at each temperature, before the DLS measurements at an angle of 90°.

### 2.8. Fluorescence Correlation Spectroscopy

Fluorescence correlation spectroscopy (FCS) measurements of the 1:1 and 2:1 SRP:DNA ROX mixtures were obtained with a Leica TCS SP5 laser scanning microscope (Wetzlar, Germany) using an HCX PL APO CS 1.20 W 63× water immersion objective, with an ISS VISTA correlator and software (Leica, Wetzlar, Germany). The correlation curves were analyzed with the 3D-Gaussian ratio model. All the samples were prepared using deionized water. To determine the calibration parameters of the system (*w*_0_ and *z*_0_/*w*_0_), we used a rhodamine 6G (R6G, Sigma-Aldrich, St. Louis, MO, USA) solution (5 × 10^−7^ M) with a diffusion coefficient of 4.14 × 10^−6^ cm^2^/s at 25 °C [26]. The diffusion coefficients for the mixtures containing DNA-ROX were obtained by global fitting of several autocorrelation curves (Supplementary Materials, Figure S4). Hydrophobic, uncoated and sterile 18 wells μ-Slides from ibidi GmbH were used in the measurements.

### 2.9. UV–Vis Absorption Spectroscopy

UV–Vis absorption spectra were measured in a Jasco V-660 spectrophotometer (Oklahoma City, OK, USA) equipped with Peltier temperature control module. Absorption spectra were recorded between 350 and 800 nm. The solutions were slowly heated at a rate of 0.5 °C/min from 30 to 56 °C, with an interval of (2 ± 0.1) °C between measurements. The solutions equilibrated for 4 min at each temperature.

### 2.10. Fluorescence Spectroscopy

Fluorescence measurements were recorded on a Horiba Jobin Yvon Fluorolog 3-22 spectrofluorometer (Kyoto, Japan) equipped with an F-3004 Peltier temperature control module. Emission spectra were obtained between 600 and 750 nm, by excitation at 585 nm, at right angle geometry. The solutions were equilibrated for 8 min at each temperature, from 32 to 54 °C, and the measurements were performed with a temperature interval of (2 ± 0.2) °C.

## 3. Results and Discussion

### 3.1. RAFT Cationic Stimuli-Responsive Copolymer

The cationic temperature-responsive copolymer was prepared by sequential RAFT polymerization, with a first temperature-responsive block composed by a mixture of the monomers 2-(2′-methoxyethoxy)ethyl methacrylate (DEGMA, **2**, Figure 1) and oligo(ethylene glycol) methacrylate (OEGMA, **3**, Figure 1). The second block was prepared by adding a cationic monomer, trimethyl-2-methacroyloxyethylammonium chloride (TMEC, **5**, Figure 1). Based on the amount of monomers used we expected a molecular weight of 29,120 g/mol (119 DEGMA monomers and 13 OEGMA monomers) for the first block and 11,835 g/mol for the cationic block (57 TMEC monomers), corresponding to a molecular weight for 100% conversion of 40,955 g/mol. The experimental molecular weight of the temperature-responsive block, obtained by NMR, was 20,500 g/mol, corresponding to 81 DEGMA monomers and 9 OEGMA monomers, while the molecular weight of the copolymer obtained by elemental analysis was 28,400 g/mol, based on the mass percentages of carbon (53.7%), hydrogen (8.4%) and nitrogen (1.9%). This value was confirmed by GPC, which yielded a polydispersity index of 1.19, typical of controlled RAFT polymerization. The molecular weight of the second block is thus 7900 g/mol, corresponding to 38 monomers of TMEC.

**Figure 2:**
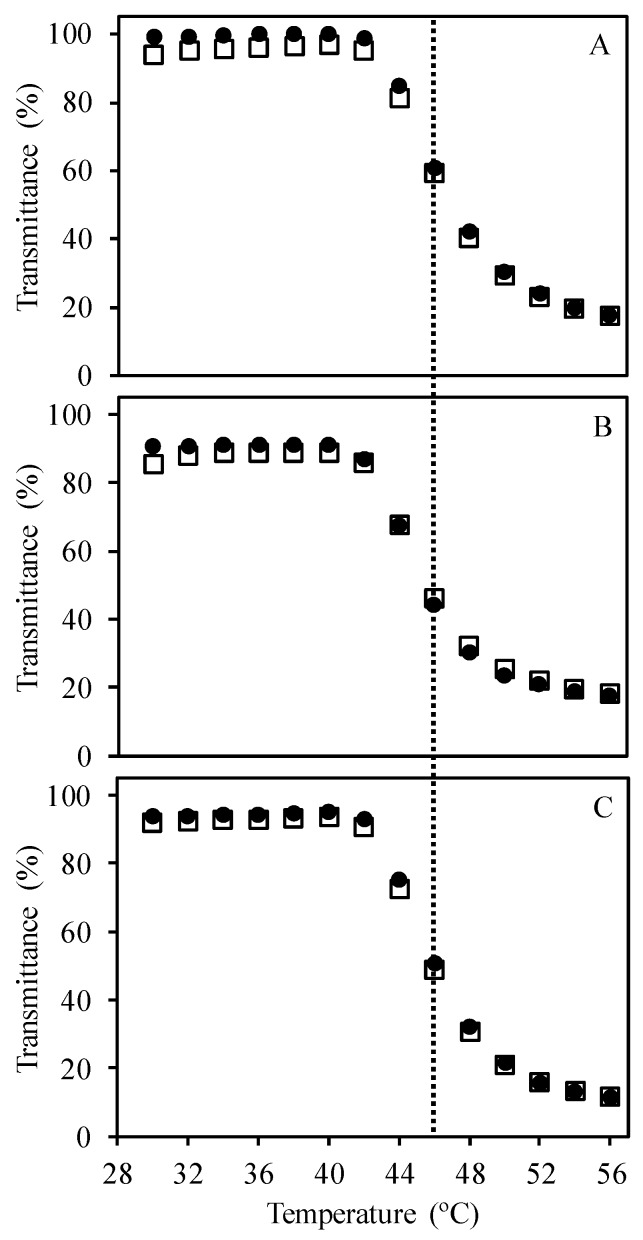
Variation of transmittance (at 400 nm) with temperature for heating (circles) and cooling (squares) cycles for (**A**) block copolymer chains in water, and for mixtures of (**B**) 1:1 SRP:DNA and (**C**) 2:1 SRP:DNA in water. Samples in (**B**,**C**) have a SRP concentration of 1 × 10^−5^ M. The dashed lines represent the volume phase transition temperature, *T*_VPT_ = 46 °C.

The temperature response of the SRP in water was studied by measuring the transmittance of an aqueous solution at 400 nm, with heating/cooling cycles between 30 and 56 °C (Supplementary Material, Figure S1A,B). The increase in temperature induces a volume phase transition with the polymer chains changing from an expanded conformation below their volume phase transition temperature (*T*_VPT_) to a collapsed conformation above this temperature. The collapse of the copolymer induces flocculation that originates light scattering (with lower transmittance detected). The measured volume phase transition temperature (*T*_VPT_) of the block copolymer chains was 46 °C, with no hysteresis on heating/cooling cycles (Figure 2A). This value is close to the reported value of the *T*_VPT_ for copolymers with the same OEGMA/DEGMA ratio (42 °C) [23], with the difference between the two values probably related to the presence of the charged TMEC block [11].

### 3.2. Interaction of the Block Copolymer Chains with a Fluorescently-Labeled Oligonucleotide

**Figure 3:**
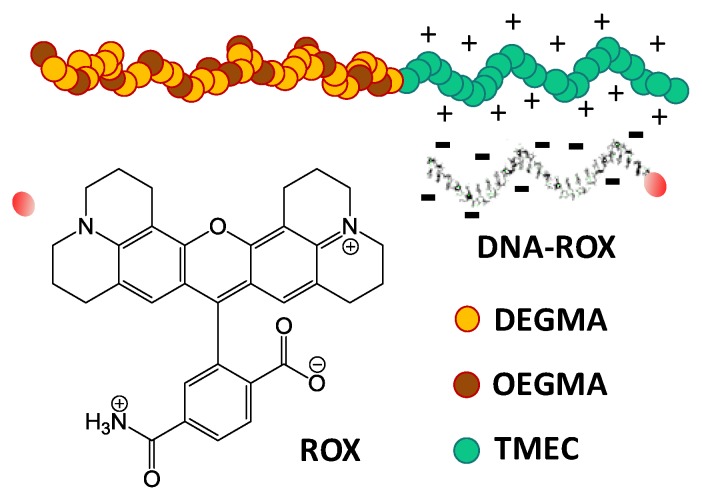
Schematic representation showing the relative scale of the stimuli-responsive block copolymer chain (SRP) and the poly-thymine oligonucleotide labelled with rhodamine X (DNA-ROX).

The model single strand DNA sequences, composed by 25 thymine units (dT_25_), are expected to form coacervated structures with the charged block of the copolymers (Figure 3). We first verify that the thermoresponsive behavior of the SRP was maintained in the coacervated structure with the DNA, as shown by the similarity of the transmission plots of the polymer solution (Figure 2A) and the SRP:DNA 1:1 and 2:1 mixtures (Figure 2B,C; transmission spectra in Figures S2 and S3, Supplementary Material).

**Figure 4:**
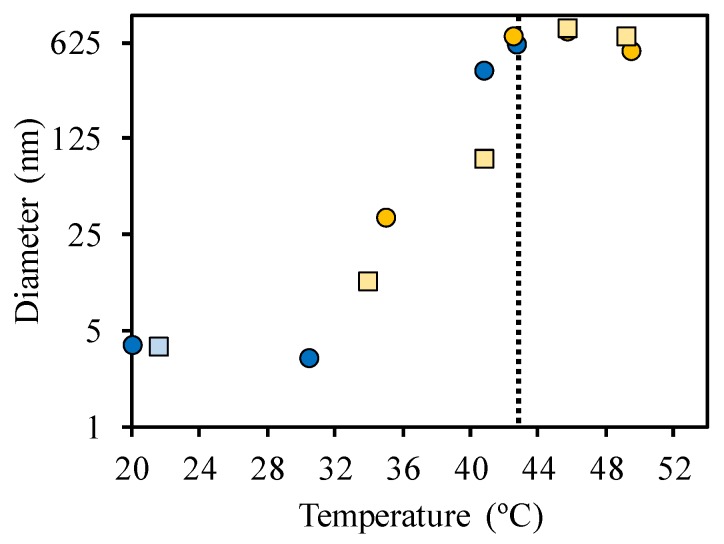
Variation of hydrodynamic diameter (measured by dynamic light scattering, DLS) with temperature for block copolymer chains in water for heating (blue circles) and cooling (blue square) cycles, and for the mixture of 1:1 SRP:DNA for heating (orange circles) and cooling (orange squares) cycles. The SRP concentrations were 2 × 10^−5^ M and 1 × 10^−5^ M for the SRP solution and 1:1 SRP:DNA mixture, respectively. The dotted line represents the *T*_VPT_ (43 °C).

In order to characterize the structures of the SRP-DNA coacervates formed in water, we measured the DLS hydrodynamic diameters of the SRP and of the 1:1 SRP:DNA mixtures at different temperatures (Figure 4). For the block copolymer chains, the hydrodynamic diameter at 20 °C is *D*_h_ = 3.8 ± 0.6 nm. By increasing the temperature, the copolymer starts to aggregate at around 43 °C, close to its *T*_VPT_ (Figure 4, blue circles). The aggregation is reversible, with the diameter of isolated chains (*D*_h_ = 3.8 nm) being recovered by cooling the sample to 22 °C (Figure 4, blue square). The 1:1 SRP:DNA mixture (Figure 4, orange circles), has a hydrodynamic diameter of *D*_h_ = 35 ± 6 nm at 35 °C (below the *T*_VPT_ of the SRP), which also increases with temperature due to aggregation. Similarly, the diameter decreases upon cooling (Figure 4, orange squares) following the same heating profile to reach the molecular diameter of the coacervate at 34 °C. 

For the 2:1 SRP:DNA mixture, the hydrodynamic diameter obtained was *D*_h_ = 45 ± 8 nm, at 30 °C (below the *T*_VPT_ of the SRP). Although the hydrodynamic diameter is slightly larger in this case, the transmission plots obtained for 1:1 and 2:1 SRP:DNA mixtures are very similar, indicating that the same type of structure is probably formed in both cases.

**Figure 5:**
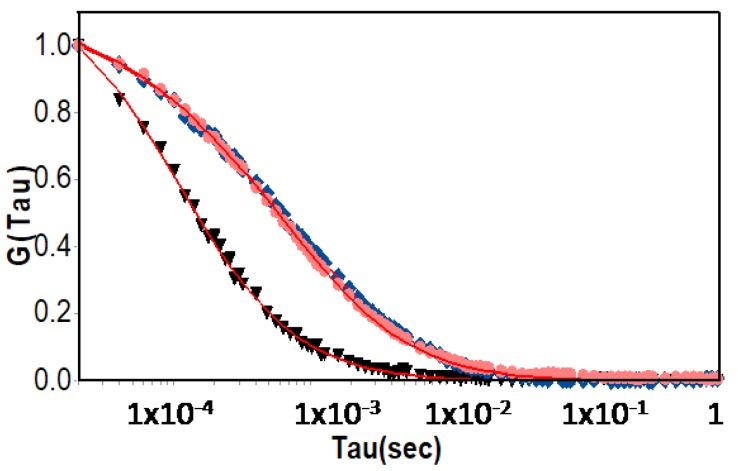
Auto-correlation curves obtained for DNA-ROX (black inverted triangles) and for the mixtures of 1:1 (blue diamonds) and 2:1 (red circles) of SRP:DNA-ROX, using fluorescence correlation spectroscopy.

To confirm the formation of SRP:DNA coacervates at low temperature (below the *T*_VPT_ of the SRP), we used fluorescence correlation spectroscopy (FCS), that allows us to discriminate between free oligonucleotides in solution and those forming coacervated complexes with the block copolymer. Since the technique measure the diffusion coefficients of the fluorescent species only, there is no interference of the non-fluorescent free copolymer. Previously characterized dT_25_ oligonucleotides labeled at the 5′-terminus with a rhodamine X [27,28,29] showed that the probe has a high quantum yield and good photostability. FCS measurements of the 1:1 and 2:1 SRP:DNA-ROX mixtures yield the diffusion coefficients of the samples, from which the hydrodynamic radius (*R*_FCS_) can be calculated using the Stokes-Einstein equation. In Figure 5, we represent the average auto-correlation curves for DNA-ROX (black inverted triangles) and for the mixtures of 1:1 (blue diamonds) and 2:1 (red circles) of SRP:DNA-ROX (from different five measurements of each sample, Figure S4, Supplementary Material). The diffusion coefficient obtained for DNA-ROX was 98.24 μm^2^/s, corresponding to a hydrodynamic radius *R*_FCS_ = 2.2 nm. The length of the relatively rigid dT_25_ oligonucleotides is estimated as ca. 8 nm (both by MD simulations and from the statistical monomer length) [27,30], corresponding to a gyration radius *R*_G_ = 2.3 nm (calculated for a rod-shaped object), which is close to the value obtained by FCS.

The shape of the FCS correlation curves for the 1:1 and 2:1 SRP:DNA-ROX mixtures are very similar (Figure 5) and can only be fitted with two component decays, attributed to two fluorescent species with different diffusion coefficients. Since a small amount of free DNA-ROX is expected, we fixed its correlation time and so the diffusion coefficient of the coacervates of DNA-ROX and SRP can be obtained with higher precision. Diffusion coefficients of ca. 10 μm^2^/s were obtained for both 1:1 and 2:1 SRP:DNA-ROX mixtures, corresponding to a hydrodynamic diameter *D*_FCS_ = 44 nm (Table 1). This indicates that the coacervated dimensions are similar for both mixtures and that they incorporate a large number of SRP chains and oligonucleotides. The excess polymer in the 2:1 SRP:DNA-ROX mixture, relative to the 1:1 SRP:DNA-ROX, seems to have little influence on the DNA-SRP coacervate aggregates at temperatures below the *T*_VPT_ because, although the hydrodynamic diameter determined by DLS increases, this increase is not detected by FCS.

**Figure 6:**
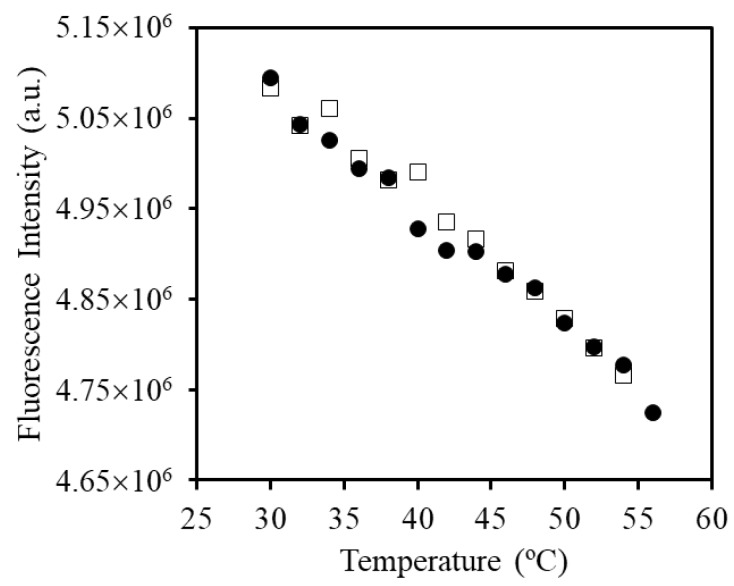
Emission intensity, measured at 610 nm for temperatures between 30 and 56 °C of DNA-ROX (1 × 10^−6^ M) by excitation at *λ*_exc_ = 585 nm. The heating and cooling cycles are represented by circles and squares, respectively.

To better understand the interaction between the block copolymer chains and the oligonucleotides, we measured the fluorescent intensity of the ROX-labelled DNA strands in the adducts, as a function of temperature. The fluorescence intensity of the DNA-ROX (1 × 10^−6^ M) in water as a function of temperature depends linearly and reversibly on the temperature (Figure 6), due to the variation of the fluorescence quantum yield with temperature.

**Figure 7:**
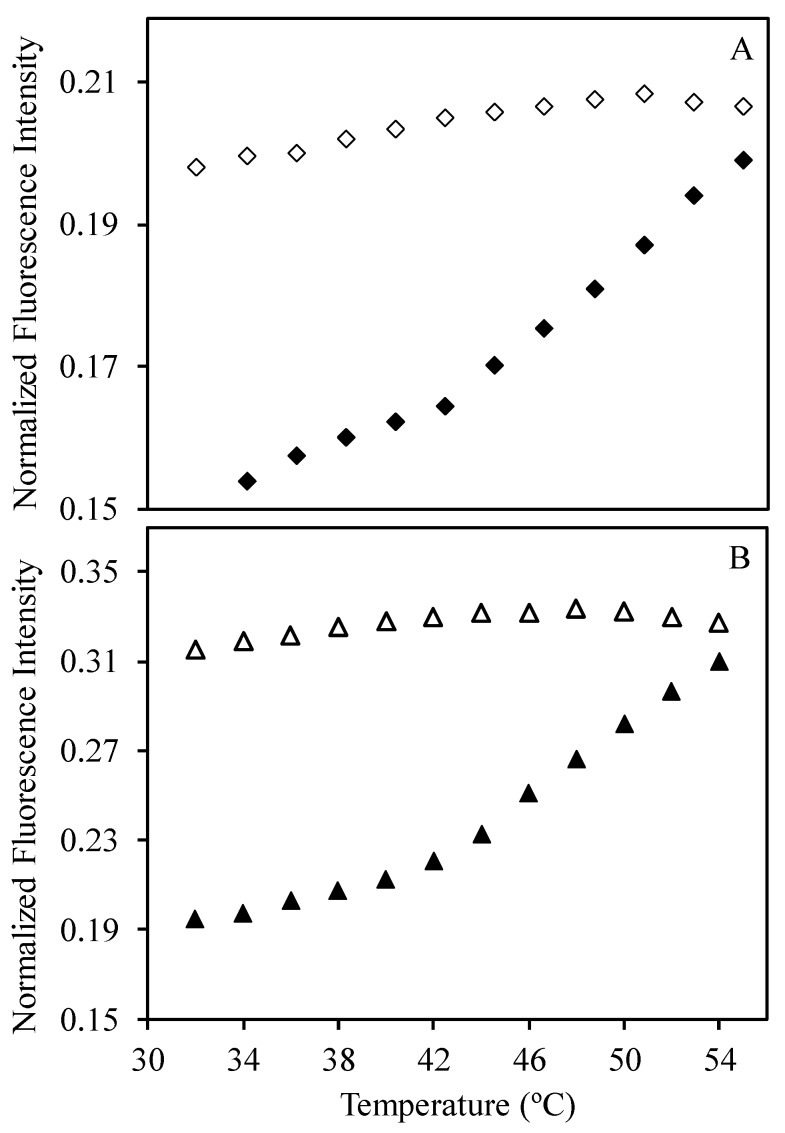
Fluorescence intensity of (**A**) 1:1 and (**B**) 4:1 SRP:DNA-ROX mixtures, measured at 610 nm for temperatures between 32 and 54 °C, normalized for the change in ROX quantum yield with temperature. The heating and cooling cycles are represented by full and empty symbols, respectively.

Fluorescence measurements for 1:1, 2:1, and 4:1 mixtures of SRP and DNA-ROX (with 5 × 10^−6^ M of DNA-ROX for all samples), at temperatures between 32 and 54 °C (Figures S5–S8, Supplementary Material) were corrected for the variation of ROX quantum yield, using the DNA-ROX fluorescence measurements obtained in the same conditions (Figure 6). In Figure 7, we show the corrected emission intensity profiles of 1:1 and 4:1 SRP:DNA-ROX mixtures. A change in the slope of the heating curve is observed in the corrected fluorescence intensity at ca. 42 °C, showing that the conformational transition of the SRP chains at the *T*_VPT_ (from a coiled to a collapsed state), induces an increase in the ROX emission intensity. This is probably caused by the disruption of (non-emissive) ROX dimers or aggregates that are present at low temperatures. When the temperature is subsequently decreased, the fluorescence intensity does not return to its initial value, indicating that the collapse and expansion of the SRP chains effectively prevent the re-aggregation of the ROX. The normalized fluorescence intensity obtained for the 1:4 SRP:DNA-ROX mixture is approximately 30% higher than for the 1:1 SRP:DNA-ROX mixture, showing that the excess SRP reduces fluorescence quenching of the ROX, and thus prevents the aggregation of the ROX-labelled DNA strands in the coacervated structures.

**Figure 8:**
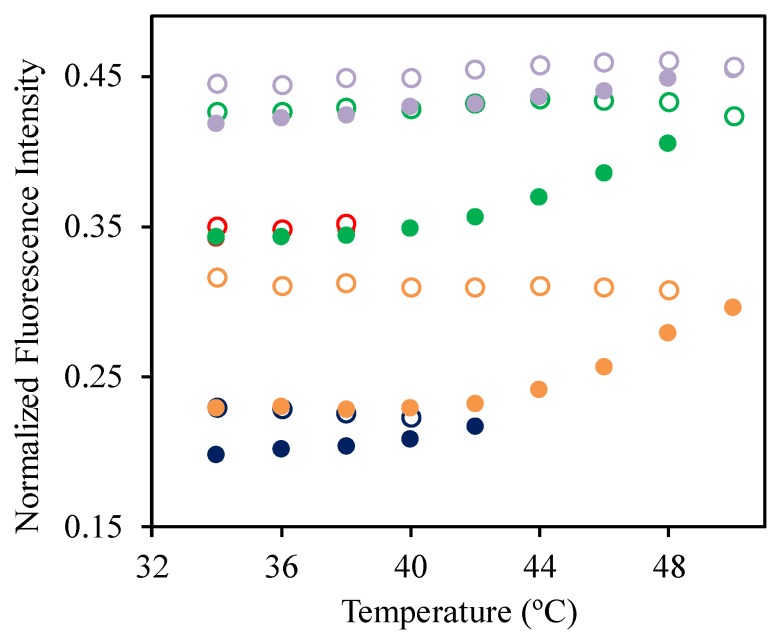
Normalized fluorescence intensity of the 2:1 SRP:DNA-ROX mixture, measured at 610 nm for temperatures between 34 and 50 °C. The mixture was heated (full circles) and cooled (empty circles) several times, following the order blue-orange-red-green-violet. The concentration of DNA-ROX is 5 × 10^−6^ M. The normalized fluorescence intensity at 610 nm was obtained by dividing the fluorescence intensity of the mixture by the fluorescence intensity of DNA-ROX obtained in the same condition, to correct for the change in ROX quantum yield with temperature.

To better understand the effect of the SRP conformational changes on the aggregation of the ROX-labeled DNA strands, we performed several successive heat/cooling cycles on the 2:1 SRP:DNA-ROX mixture (Figure 8). In the first cycle, we heated the sample from 34 to 42 °C observing an increase in normalized fluorescence intensity, similar to what was observed for the 1:1 and 1:4 mixtures (Figure 7, full blue circles). Upon cooling to 34 °C, the fluorescence intensity did not return to the initial values, also as observed for the 1:1 and 1:4 mixtures (Figure 7, empty blue circles). 

In the second cycle (Figure 7, full orange circles), the sample was heated up to 50 °C and cooled down to 34 °C, again without loss of the fluorescence intensity during cooling. This indicates that, once the ROX-labeled DNA strands are disaggregated by the collapse of the SRP into the globular form, DNA-ROX reaggregation is prevented by the SRP, even after the transition to the expanded coil conformation. In the third cycle, the sample was heated only to 38 °C (Figure 7, full red circles, bellow *T*_VPT_) and then cooled back to 34 °C (Figure 7, empty red circles). In this case, there is no hysteresis in the fluorescence intensity, showing that the conformational transition in the polymer is responsible for preventing re-aggregation of the dye during cooling. 

Heating the sample to 50 °C and cooling to 34 °C (green circles), the normalized fluorescence intensity still increased (without decreasing upon cooling), but when this process was repeated a fifth time (violet circles), no hysteresis was observed, with the same normalized fluorescence intensity being recovered upon heating and cooling. This means that the sample reached its maximum emission intensity, which we interpret as meaning that no further DOX aggregation is present, with all DNA-ROX well separated within the coacervated structures.

In conclusion, the conformation changes in the temperature responsive block effectively separate the DNA strands, preventing ROX fluorescence quenching, so that the strands are individually stabilized within the DNA-SRP coacervated structures.

**Figure 9:**
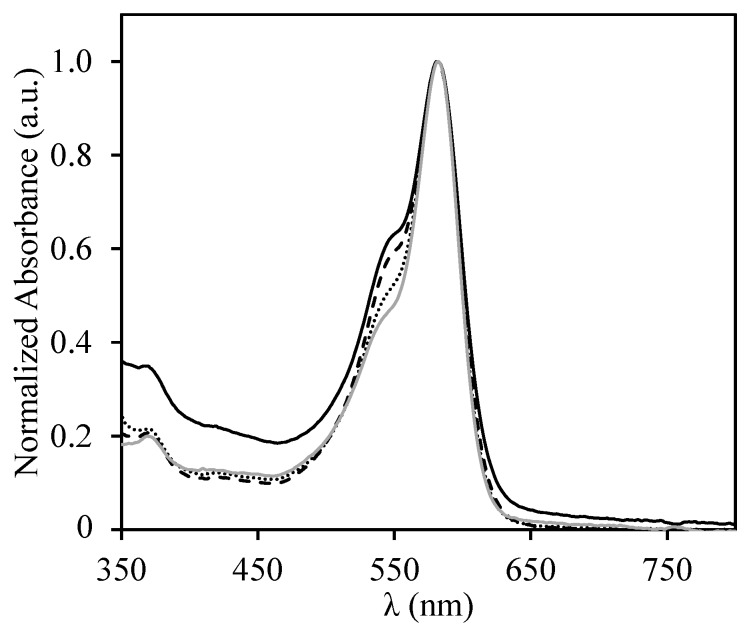
Normalized absorption spectra for DNA-ROX (grey solid line) and for the mixtures of 1:1 (black solid line), 2:1 (dashed line), and 4:1 (dotted line) of SRP:DNA-ROX. All samples have a DNA-ROX concentration of 5 × 10^−6^ M and different SRP concentrations.

The stabilization of the DNA-ROX strands by the copolymer can be confirmed by comparing the absorption spectra of the 1:1, 2:1, and 4:1 SRP:DNA-ROX mixtures (Figure 9). The formation of ROX dimers in water is unfavorable due to electrostatic repulsions between the DNA strands. However, when the coacervates with the SRP are formed, partial neutralization of the charges leads to the formation of ROX dimers (visible by the appearance of a shoulder in the absorption band at around 550 nm), that indicates DNA strand aggregation. The increase in the amount of polymer in the mixtures probably leads to an excess positive charge in the coacervates that reduces the formation of ROX dimers (almost inexistent for the 4:1 SRP:DNA-ROX mixtures, for which the spectra is very similar to that of DNA-ROX in water—compare the grey solid line and the dotted line in Figure 9).

## 4. Conclusions

Our new smart block copolymer, composed of one temperature-responsive block and one cationic block, can bind single strand DNA chains, offering good prospects for biomedical applications, for example as a vehicle for gene delivery. The copolymer was synthesized by RAFT polymerization to guaranty size and composition homogeneity. The first block is composed by di(ethylene glycol) methyl ether methacrylate (DEGMA) and an oligo(ethylene glycol) methyl ether methacrylate (OEGMA), with a DEGMA/OEGMA ratio that gives a volume phase transition temperature in water of 46 °C. This temperature can be changed over a wide range by controlling the DEGMA/OEGMA ratio in the block. The second block of trimethyl-2-methacroyloxyethylammonium chloride (TMEC) is positively charged at physiological pH values and was used to bind a model DNA strand. The resulting coacervate complexes are formed through strong electrostatic interactions between several DNA strands and copolymer chains, and can withstand temperature variations inducing the collapse/expansion of the polymer chain. These conformational changes in the temperature-responsive block can separate the DNA strands in these multi-component complexes. 

The combination of dynamic light scattering with fluorescence correlation spectroscopy (FCS) and fluorescence spectroscopy allowed us to probe the nature of the DNA-SRP coacervate complexes and understand the effect of the temperature-induced polymer phase transition on the structure of the complexes, leading to a temperature-cycling procedure that can effectively separate the DNA strands in these structures.

## Figures and Tables

**Table 1:** Room temperature hydrodynamic diameters obtained for DNA-ROX, SRP and for the 1:1 and 2:1 mixtures of SRP:DNA-ROX, using dynamic light scattering (DLS) and fluorescence correlation spectroscopy (FCS).

Sample	Hydrodynamic Diameter (nm)	
	DLS	FCS
SRP	3.8 ± 0.6	-
DNA-ROX	-	4.4 ± 0.4
1:1 SRP:DNA-ROX mixture	35 ± 6	44 ± 5
2:1 SRP:DNA-ROX mixture	45 ± 8	44 ± 5

## Supplementary Materials

The following are available online at http://www.mdpi.com/2073-4360/9/11/576/s1. Figure S1: Transmittance vs temperature for an SRP solution; Figure S2: Transmittance vs. temperature for a mixture of 1:1 SRP:DNA-ROX; Figure S3: Transmittance vs temperature for a mixture of 2:1 SRP:DNA-ROX ; Figure S4: Auto-correlation curves obtained for DNA-ROX, and for the mixtures of 1:1 and 2:1 of SRP:DNA-ROX, using fluorescence correlation spectroscopy; Figure S5: Fluorescence intensity for a range of temperatures between 32 and 54 °C for mixtures of 1:1, 2:1, and 4:1 SRP:DNA-ROX; Figure S6: Emission spectra for the mixture of 1:1 SRP:DNA-ROX ; Figure S7: Emission spectra for the mixture of 4:1 SRP:DNA-ROX ; Figure S8: Emission spectra for the mixture of 2:1 SRP:DNA-ROX.
