# Supplementary material for: A Cationic Smart Copolymer for DNA Binding
Source: Polymers (Basel). 2017 Nov 4;9(11):576. doi: 10.3390/polym9110576 (PMC6418539; doi:10.3390/polym9110576)
Supplement: Supplementary file 1 [file polymers-09-00576-s001.pdf]

Supplementary Information

A cationic smart copolymer for DNA binding

Tânia Ribeiro, Ana M. Santiago, José M. G. Martinho, José Paulo S. Farinha *

CQFM – Centro de Química-Física Molecular and IN – Institute of Nanoscience and Nanotechnology, Instituto Superior Técnico, Universidade de Lisboa, 1049-001 Lisboa, Portugal.

* Corresponding author: Tel.: +351 218419221; E-mail: farinha@tecnico.ulisboa.pt

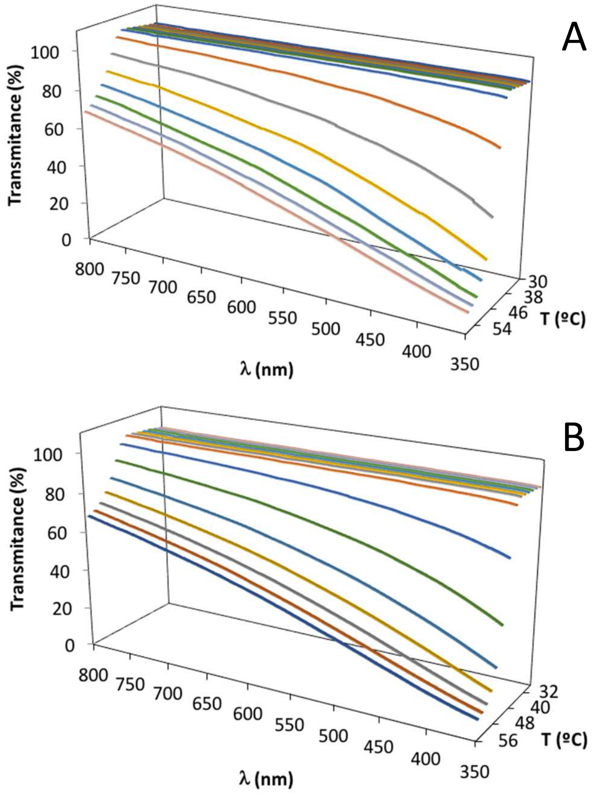

Figure S1. Transmittance vs temperature for a SRP solution ([SRP]= 1×10^{-5} M), **(A)** increasing the temperature from 30 to 56 ºC and **(B)** decreasing the temperature from 56 to 30 ºC.

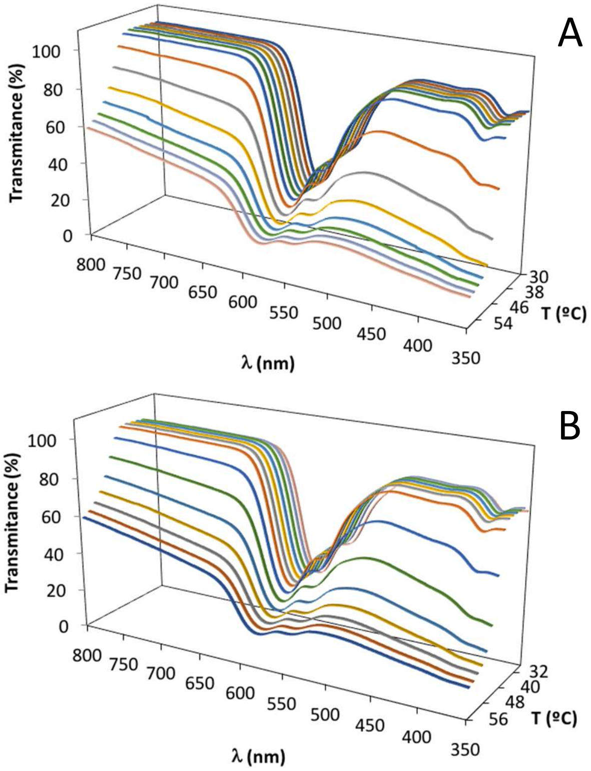

Figure S2. Transmittance vs temperature for a mixture of 1:1 SRP:DNA-ROX ([SRP]= 1×10^{-5} M), **(A)** increasing the temperature from 30 to 56 ºC and **(B)** decreasing the temperature from 56 to 30 ºC.

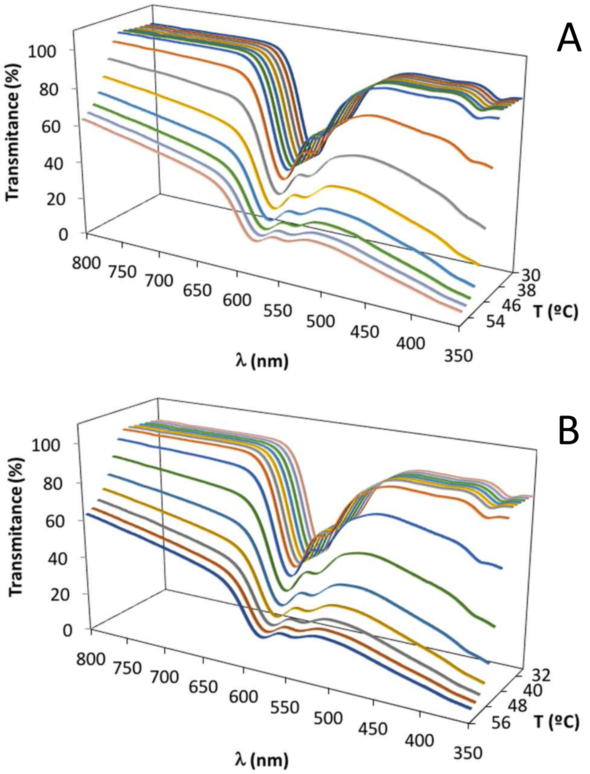

Figure S3. Transmittance vs temperature for a mixture of 2:1 SRP:DNA-ROX ([SRP]= 1×10^{-5} M), **(A)** increasing the temperature from 30 to 56 ºC and **(B)** decreasing the temperature from 56 to 30 ºC.

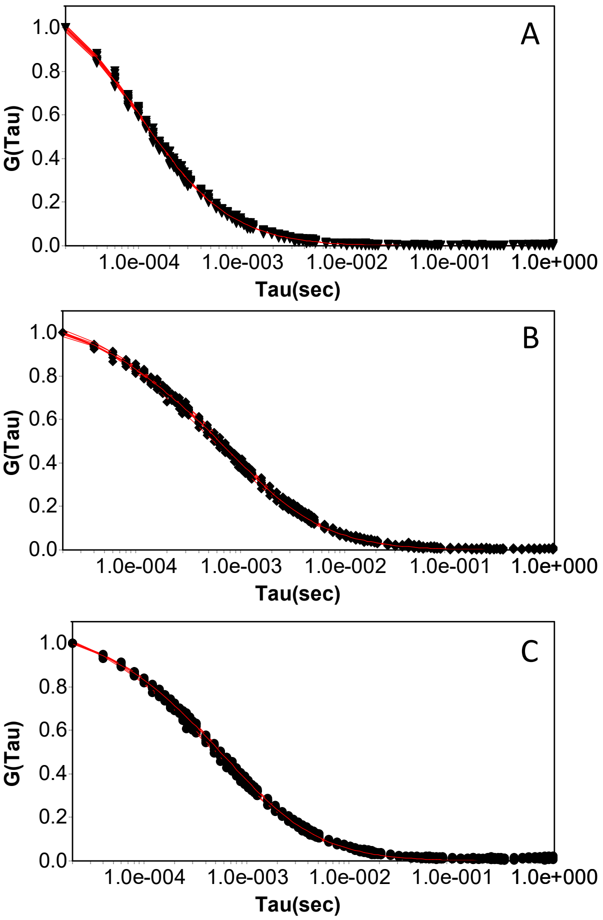

Figure S4. Auto-correlation curves obtained for DNA-ROX (A) and for the mixtures of 1:1 (B) and 2:1 (C) of SRP:DNA-ROX, using fluorescence correlation spectroscopy.

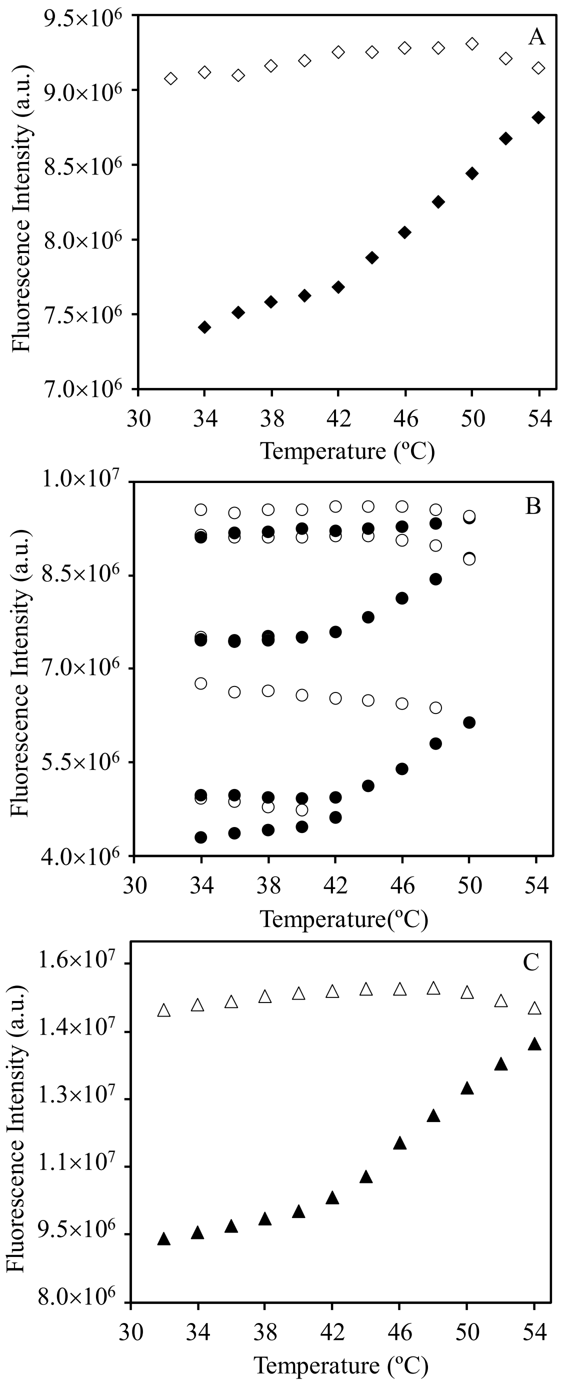

Figure S5. Fluorescence intensity, measured at 610 nm for a range of temperatures between 32 and 54 ºC for mixtures of (A) 1:1, (B) 2:1 and (C) 4:1 of SRP:DNA-ROX. The mixtures were warmed (full markers) and cooled (bare markers). The concentration of DNA-ROX was 5×10^{-6} M for all the samples.

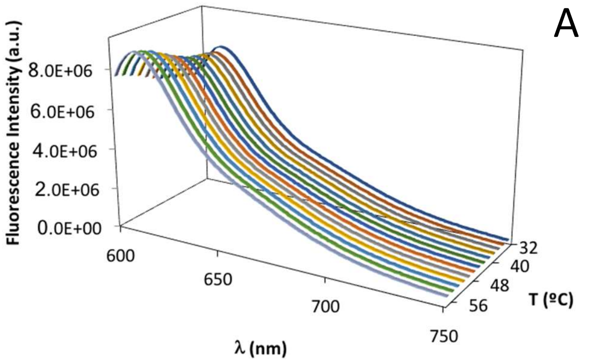

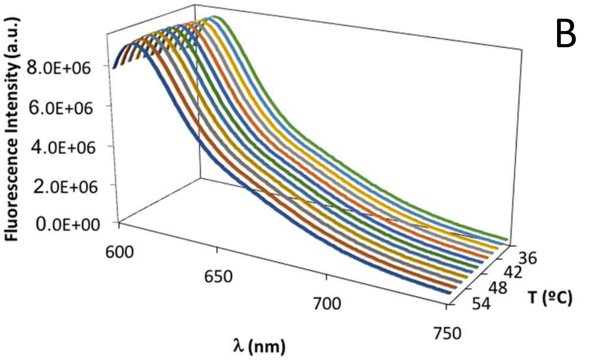

Figure S6. Emission spectra using λ_{exc} = 585 nm, for the mixture of 1:1 SRP:DNA-ROX (5×10^{-6} M) **(A)** increasing the temperature from 32 to 56 ºC and **(B)** decreasing the temperature from 54 to 32 ºC.

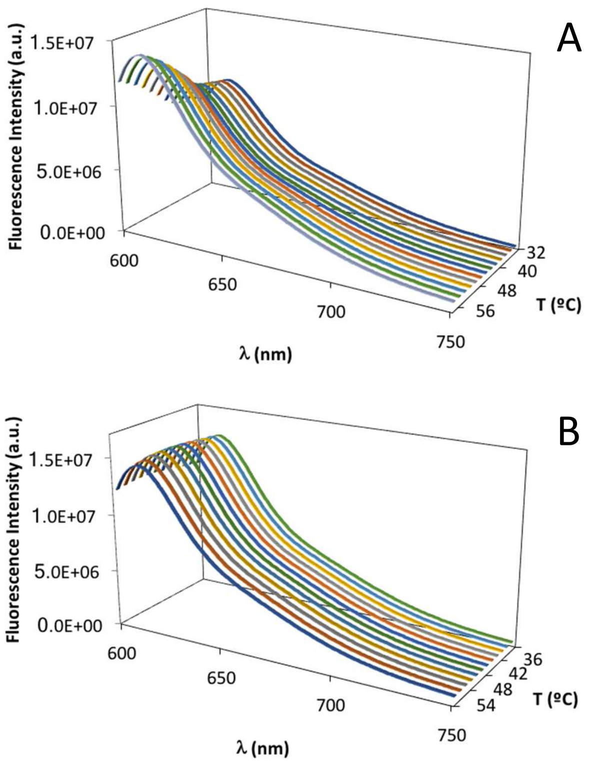

Figure S7. Emission spectra using λ_{exc} = 585 nm, for the mixture of 4:1 SRP:DNA-ROX (5×10^{-6} M) **(A)** increasing the temperature from 32 to 56 ºC and **(B)** decreasing the temperature from 54 to 32 ºC.

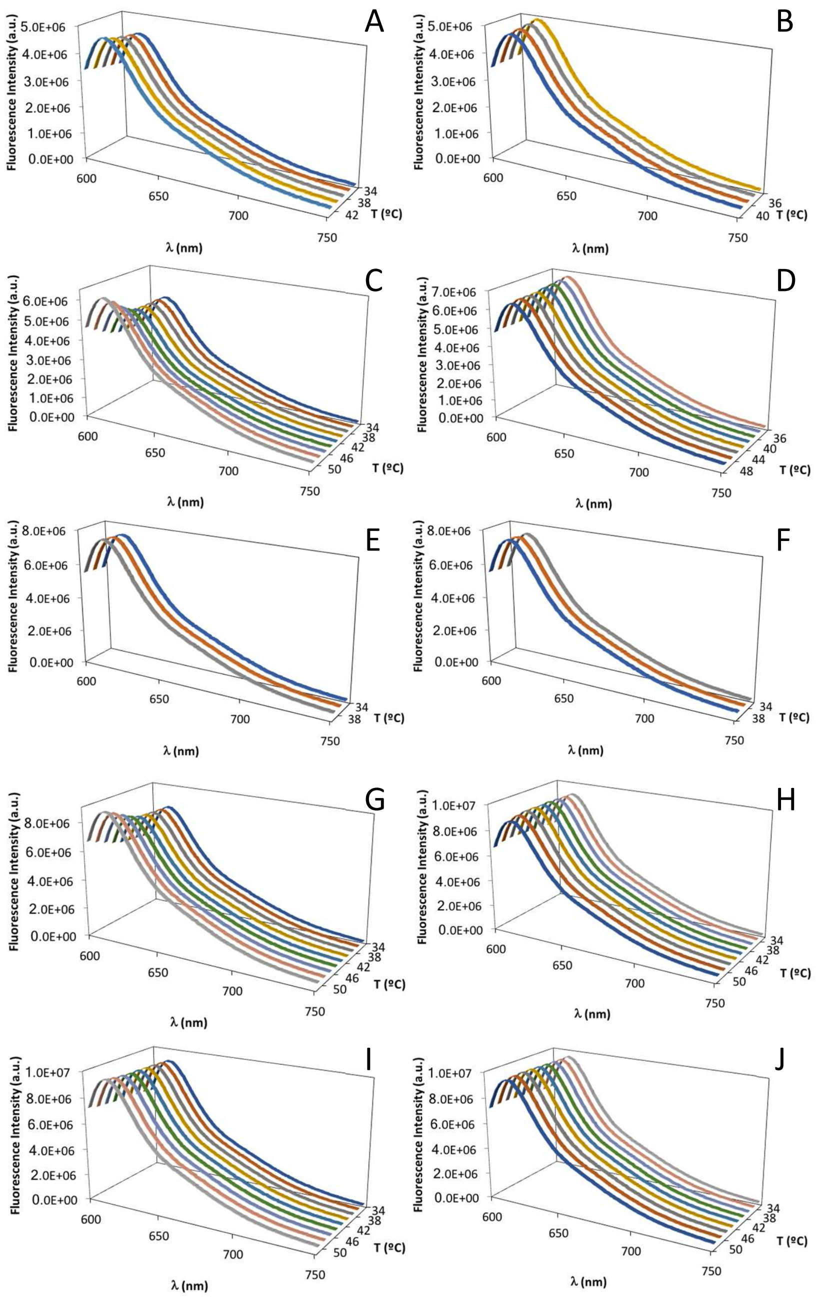

Figure S8. Emission spectra using λ_{exc} = 585 nm, for the mixture of 2:1 SRP:DNA-ROX (5×10^{6} M) **(A)** increasing the temperature from 34 to 42 ºC (first heating), **(B)** decreasing the temperature from 40 to 34 ºC, **(C)** increasing the temperature from 34 to 50 ºC (second heating), **(D)** decreasing the temperature from 50 to 34 ºC, **(E)** increasing the temperature from 34 to 38 ºC (third heating), **(F)** decreasing the temperature from 38 to 34 ºC, **(G)** increasing the temperature from 34 to 50 ºC (fourth heating), **(H)** decreasing the temperature from 50 to 34 ºC, **(I)** increasing the temperature from 34 to 50 ºC (fifth heating) and **(J)** decreasing the temperature from 50 to 34 ºC.
